# Apoptotic Signaling Across Breast Cancer Subtypes and Cryoablation-Induced Tissue Injury

**DOI:** 10.3390/ijms27125174

**Published:** 2026-06-07

**Authors:** Agata Panfil, Kacper Boroń, Tomasz Sirek, Agata Sirek, Nikola Zmarzły, Michalina Wróbel, Zbigniew Wróbel, Dariusz Boroń, Piotr Ossowski, Martyna Stefaniak, Paweł Ordon, Grzegorz Wyrobiec, Wojciech Kulej, Marcin Opławski, Bogusław Opławski, Natalia Lekston, Beniamin Oskar Grabarek

**Affiliations:** 1 Collegium Medicum, WSB University, 41-300 Dąbrowa Górnicza, Poland; drtstierka@gmail.com (T.S.); agatasirek06@gmail.com (A.S.); nikola.zmarzly@wsb.edu.pl (N.Z.); wrobelmichalina@o2.pl (M.W.); zbi.wrobel@gmail.com (Z.W.); dariusz@boron.pl (D.B.); drpiotrossowski15@gmail.com (P.O.); martynastefaniakk@gmail.com (M.S.); pawelordon4@gmail.com (P.O.); wojciechkulej87@gmail.com (W.K.); lekstonnatalia@gmail.com (N.L.); bgrabarek7@gmail.com (B.O.G.); 2 Department of Plastic Surgery, Faculty of Medicine, Academia of Silesia, 40-555 Katowice, Poland; q375@gmail.com; 3 Faculty of Medicine and Health Sciences, Andrzej Frycz Modrzewski Krakow University, 30-705 Kraków, Poland; 4 Department of Gynecology and Obstetrics, TOMMED Specjalisci od Zdrowia, 40-662 Katowice, Poland; 5 Department of Gynecology and Obstetrics with Gynecologic Oncology, Ludwik Rydygier Memorial Specialized Hospital, 31-826 Kraków, Poland; 6 Department of Plastic and Reconstructive Surgery, Hospital for Minimally Invasive and Reconstructive Surgery in Bielsko-Biała, 43-316 Bielsko-Biala, Poland; 7 Medi-Lab SC, Wróbel i Wspólnicy, 58-100 Świdnica, Poland; 8 Department of Histology and Cell Pathology in Zabrze, Faculty of Medical Sciences in Zabrze, Medical University of Silesia, 41-808 Zabrze, Poland; gwyrobiec@sum.edu.pl; 9 Department of Gynecology and Obstetrics, Faculty of Medicine and Health Sciences, Andrzej Frycz Modrzewski University Kraków, 30-705 Kraków, Poland; marcin.oplawski@gmail.com (M.O.); oplawski.b@gmail.com (B.O.); 10 Faculty of Medicine, VIZJA University, 01-043 Warszawa, Poland

**Keywords:** breast cancer, apoptosis, cell death regulation, cryoablation, microRNA

## Abstract

Apoptosis maintains tissue homeostasis, and its dysregulation is closely associated with breast cancer progression and therapeutic resistance. We performed an integrative analysis of apoptosis-related signaling in breast cancer tissues across five molecular subtypes and compared these patterns with systemic apoptotic responses following cryoablation of benign fibroadenomas. Gene expression profiling was conducted using mRNA microarrays and validated by qRT-PCR and ELISA. Apoptosis pathway activity was assessed with the MSigDB HALLMARK_APOPTOSIS gene set, including intrinsic and extrinsic pathway scoring and an apoptotic balance index (ABI). MicroRNA profiling combined with in silico analyses identified potential miRNA–mRNA interactions. A progressive shift toward reduced pro-apoptotic and enhanced stress-adaptive signaling was observed with increasing tumor aggressiveness, most pronounced in triple-negative and non-luminal HER2-positive cancers. This pattern included reduced intrinsic pathway activity, decreased ABI, downregulation of pro-apoptotic genes (*BIK*, *BMF*, *TXNIP*), and upregulation of stress-associated or cytoprotective genes (*HSPB1*, *PPT1*). Several expression patterns were accompanied by overexpression of miRNAs (miR-582-5p, miR-421, miR-106b-5p, miR-20a-5p, miR-20b-5p, miR-93-5p) predicted to target apoptosis-related genes. In contrast, fibroadenoma cryoablation was associated with transient systemic modulation of apoptosis-related genes and proteins followed by gradual normalization. These findings highlight differences between apoptosis-related dysregulation in malignant tissue and regulated systemic responses following benign tissue injury, supporting pathway-level interpretation and identifying candidate molecular networks warranting further mechanistic and translational investigation.

## 1. Introduction

Apoptosis, or programmed cell death, is a highly regulated process essential for maintaining tissue homeostasis and eliminating damaged or potentially harmful cells. It involves a complex network of signaling pathways that coordinate cellular dismantling without provoking inflammation [1]. Dysregulation of apoptosis is a hallmark of cancer, enabling survival of cells that would normally be eliminated, thereby contributing to tumor initiation, progression, and therapy resistance. Two major pathways govern apoptosis: the intrinsic, mitochondria-mediated pathway, which responds primarily to intracellular stress signals, and the extrinsic, death receptor-mediated pathway, which is activated through extracellular cues. Both pathways converge on the activation of caspases, orchestrating the orderly execution of cell death. The balance between pro- and anti-apoptotic signals is critical in determining cell fate, and its disruption can shift cells toward survival or death [2,3].

Breast cancer is a heterogeneous disease comprising multiple molecular subtypes, each characterized by distinct biological behaviors, clinical outcomes, and responses to therapy. Luminal A tumors are typically estrogen receptor (ER)-positive, HER2-negative, and characterized by relatively low proliferative activity and favorable prognosis. Luminal B tumors retain hormone receptor expression but exhibit higher proliferative rates and may be HER2-positive or HER2-negative [4,5]. HER2-positive tumors are characterized by amplification or overexpression of HER2 and often display aggressive clinical behavior. Triple-negative breast cancer (TNBC), lacking ER, progesterone receptor (PR), and HER2 expression, represents a biologically heterogeneous subtype associated with high invasiveness, early recurrence, and limited targeted therapeutic options [6,7]. Alterations in apoptotic signaling are particularly relevant in aggressive subtypes, where suppression of pro-apoptotic mechanisms and/or overexpression of anti-apoptotic genes (e.g., BCL2, HSPB1) enables cancer cells to evade programmed cell death and resist conventional therapies [8]. The tumor microenvironment can further reinforce this survival advantage by providing paracrine pro-survival signals [9]. Understanding the molecular landscape of apoptosis across breast cancer subtypes is therefore essential for elucidating tumor biology and identifying potential therapeutic vulnerabilities.

Benign breast lesions, such as fibroadenomas, are common and generally non-threatening. Minimally invasive interventions, including cryoablation, have emerged as alternatives to surgical excision, enabling targeted destruction of abnormal tissue while preserving surrounding structures [10]. Cryoablation induces cell death locally, but can also trigger systemic molecular responses, including activation of apoptosis-related pathways [11]. Studying these responses provides a unique opportunity to contrast physiological, treatment-induced apoptosis with the dysregulated apoptosis observed in malignant tissue, and to investigate how pro- and anti-apoptotic mechanisms are balanced in a controlled setting.

In this study, we aimed to comprehensively characterize apoptosis-related signaling in breast cancer tissues and peripheral blood, focusing on subtype-specific differences in intrinsic and extrinsic pathways. We further explored the systemic apoptotic response following cryoablation of fibroadenomas. By integrating transcriptomic, proteomic, and miRNA analyses, we sought to identify key genes contributing to apoptosis dysregulation in malignancy and to delineate physiological mechanisms of apoptosis activation in benign breast tissue, thereby providing insights with potential translational relevance.

## 2. Results

The analyses presented below were performed using two independent clinical cohorts comprising breast cancer tissue samples and a separate fibroadenoma cryoablation cohort. Breast cancer analyses were conducted using tissue specimens obtained from 405 female patients representing five molecular subtypes (luminal A, luminal B HER2−, luminal B HER2+, non-luminal HER2+, and TNBC), together with histologically normal surgical margins, as previously described [12,13,14]. A summary of demographic and clinicopathological characteristics, including age distribution and subtype classification, is provided in Supplementary Table S1. In addition, systemic apoptotic responses following cryoablation were evaluated in a separate cohort of 34 female patients with breast fibroadenoma using longitudinal peripheral blood sampling.

### 2.1. Apoptosis Signaling Activity Across Breast Cancer Subtypes

**Figure 1:**
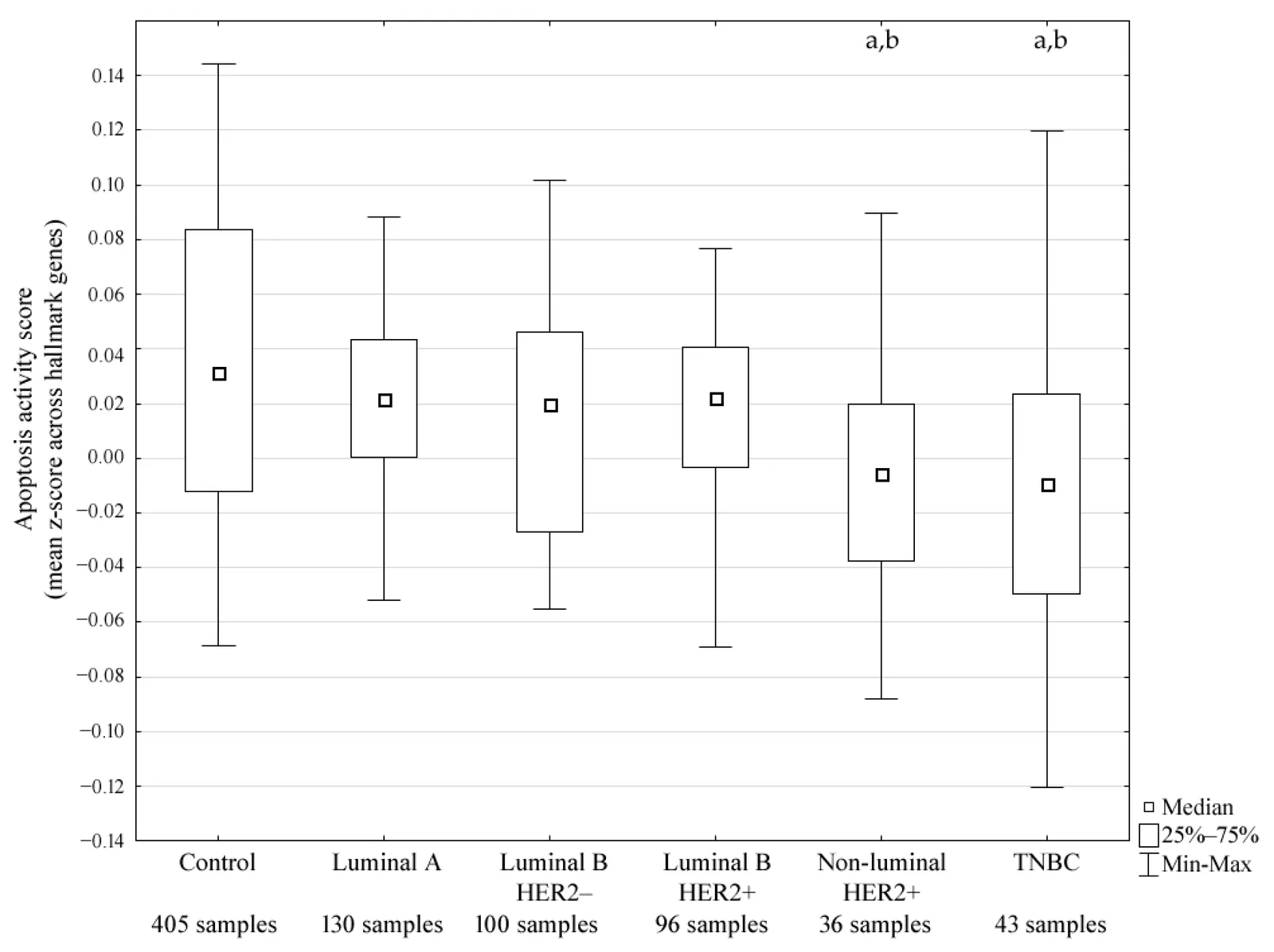
Global apoptotic activity in different breast cancer subtypes. HER2—human epidermal growth factor receptor 2; TNBC—triple-negative breast cancer. ^a^ *p* < 0.05 vs. control, ^b^ *p* < 0.05 vs. luminal A.

Using the HALLMARK_APOPTOSIS gene set from the Molecular Signatures Database (MSigDB), apoptosis pathway activity was estimated for each sample based on the mean z-score of hallmark genes. Global apoptosis activity was significantly lower in non-luminal HER2+ and TNBC samples compared to luminal A tumors and control tissue, indicating overall suppression of apoptotic signaling in the most aggressive breast cancer subtype. The distribution of apoptosis activity across breast cancer subtypes is presented in Figure 1.

To further explore whether this reduction in global apoptosis was driven by specific signaling mechanisms, intrinsic (mitochondrial) and extrinsic (death receptor-mediated) apoptotic pathway activities were calculated separately for each sample.

**Figure 2:**
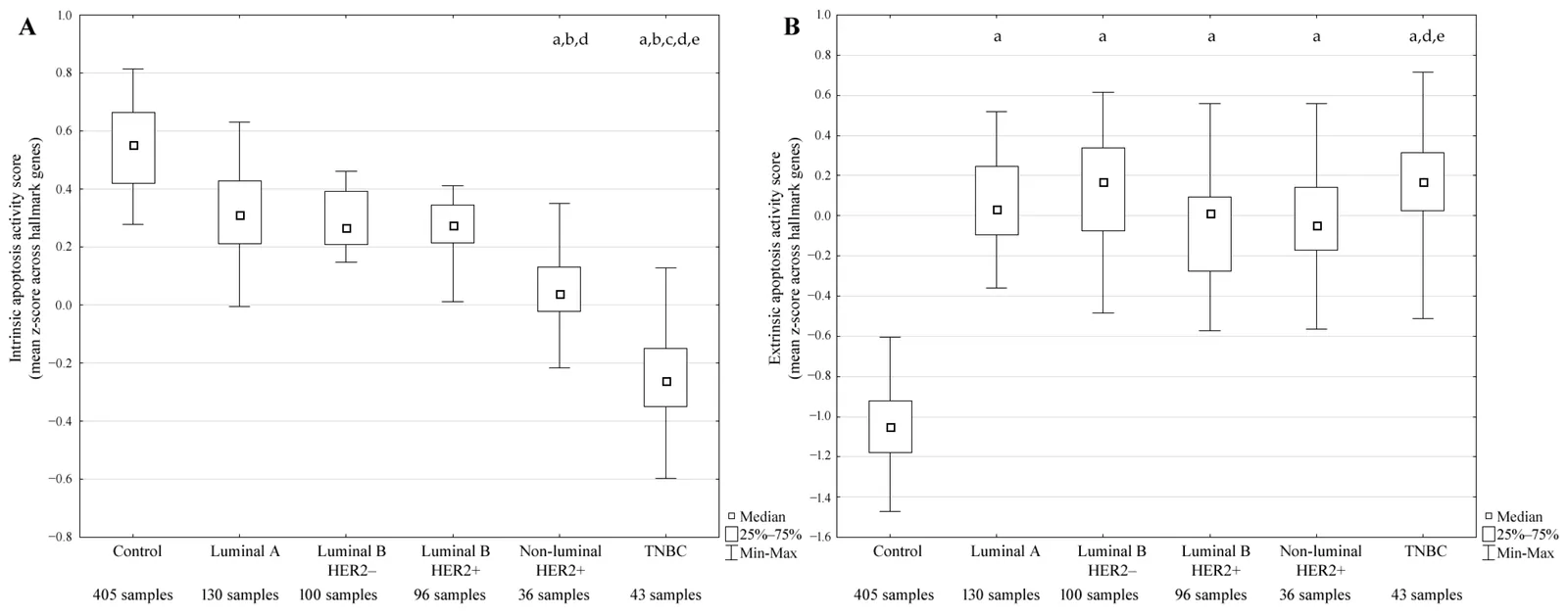
Intrinsic (**A**) and extrinsic (**B**) apoptotic pathway activity in different breast cancer subtypes. HER2—human epidermal growth factor receptor 2; TNBC—triple-negative breast cancer. ^a^ *p* < 0.05 vs. control; ^b^ *p* < 0.05 vs. luminal A cancer, ^c^ *p* < 0.05 vs. luminal B HER2−, ^d^ *p* < 0.05 vs. luminal B HER2+, ^e^ *p* < 0.05 vs. non-luminal HER2+ cancer.

Intrinsic apoptotic pathway activity progressively decreased with increasing tumor aggressiveness, reaching the lowest levels in TNBC. Additionally, non-luminal HER2-positive tumors showed significantly lower intrinsic pathway activity compared to luminal A, luminal B HER2+ and control tissues (Figure 2A). In contrast, extrinsic apoptotic signaling exhibited an opposite trend. Control tissue displayed significantly lower extrinsic pathway activity compared to all breast cancer subtypes, while TNBC showed higher extrinsic activity than luminal and non-luminal HER2-positive tumors (Figure 2B).

Detailed statistical comparisons, including *p*-values and effect size estimates with confidence intervals for all pathway-level analyses, are summarized in Supplementary Table S2.

### 2.2. Apoptotic Balance Index Analysis

**Figure 3:**
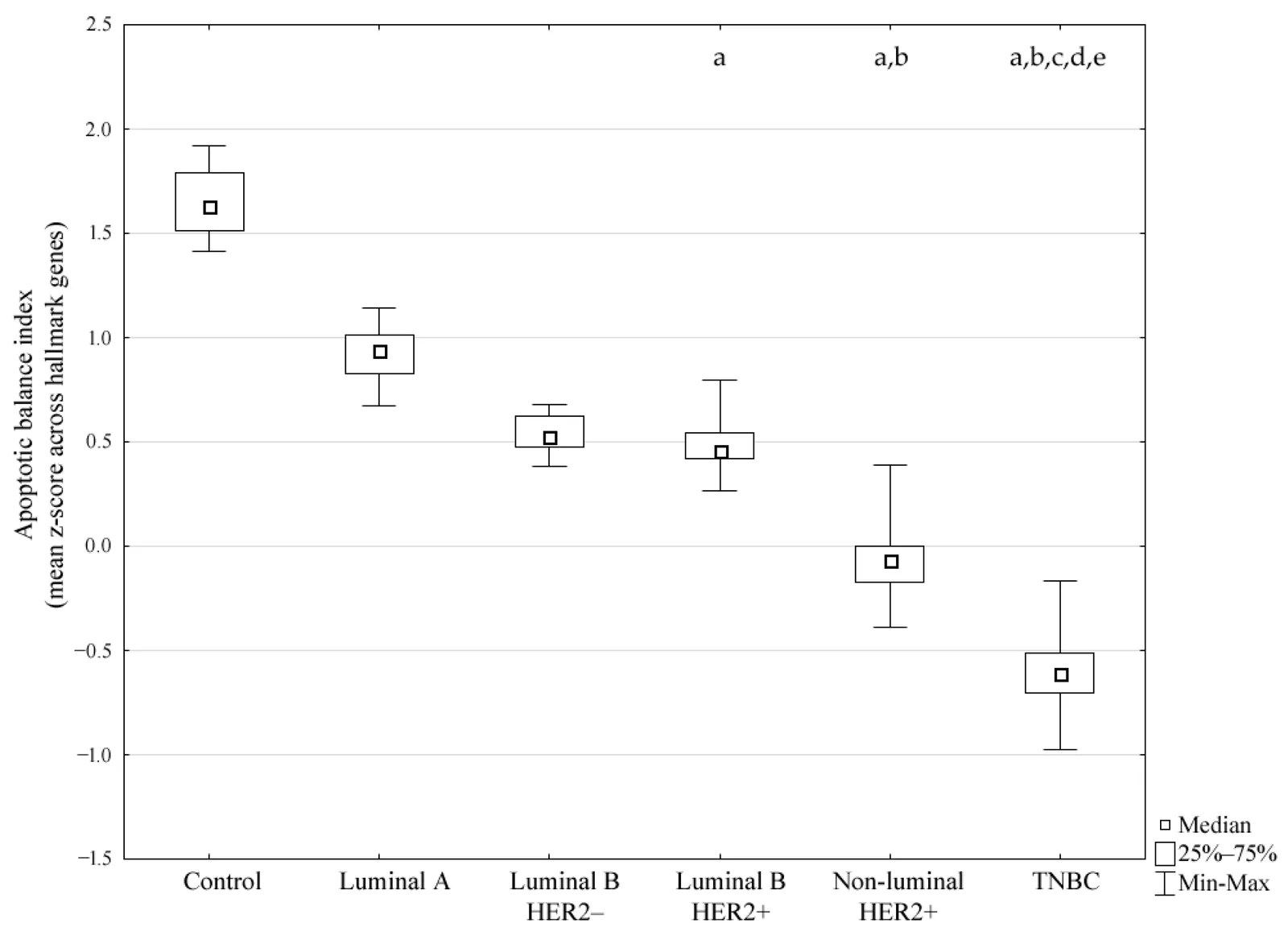
Apoptotic balance index in different breast cancer subtypes. HER2—human epidermal growth factor receptor 2; TNBC—triple-negative breast cancer. ^a^ *p* < 0.05 vs. control; ^b^ *p* < 0.05 vs. luminal A cancer, ^c^ *p* < 0.05 vs. luminal B HER2−, ^d^ *p* < 0.05 vs. luminal B HER2+, ^e^ *p* < 0.05 vs. non-luminal HER2+ cancer.

To assess the balance between pro- and anti-apoptotic signaling across breast cancer subtypes, the apoptotic balance index (ABI) was calculated based on the expression profiles of apoptosis-related genes included in the HALLMARK_APOPTOSIS gene set. ABI values were highest in control breast tissue and progressively decreased across breast cancer molecular subtypes, reaching the lowest levels in TNBC (Figure 3).

A significant reduction in ABI was observed in TNBC compared with control tissue and less aggressive subtypes, indicating a pronounced shift toward anti-apoptotic signaling in this subtype. Intermediate ABI values were detected in luminal and HER2-positive tumors, reflecting a gradual imbalance between pro- and anti-apoptotic mechanisms accompanying increasing tumor aggressiveness.

Notably, the pattern of ABI closely mirrored intrinsic apoptotic pathway activity, suggesting that suppression of mitochondrial apoptosis is a major contributor to the altered apoptotic balance observed in aggressive breast cancer subtypes. These findings indicate that, despite partial activation of extrinsic apoptotic signaling, the overall balance of apoptotic regulation is shifted toward cell survival, particularly in TNBC.

#### External Validation of Apoptotic Balance Index in the METABRIC Cohort

To evaluate the robustness and generalizability of the ABI, an independent analysis was performed using the METABRIC breast cancer cohort accessed via cBioPortal. ABI was calculated using the same gene set and computational framework as in the discovery cohort, based on z-score normalized gene expression values computed within the METABRIC dataset.

**Figure 4:**
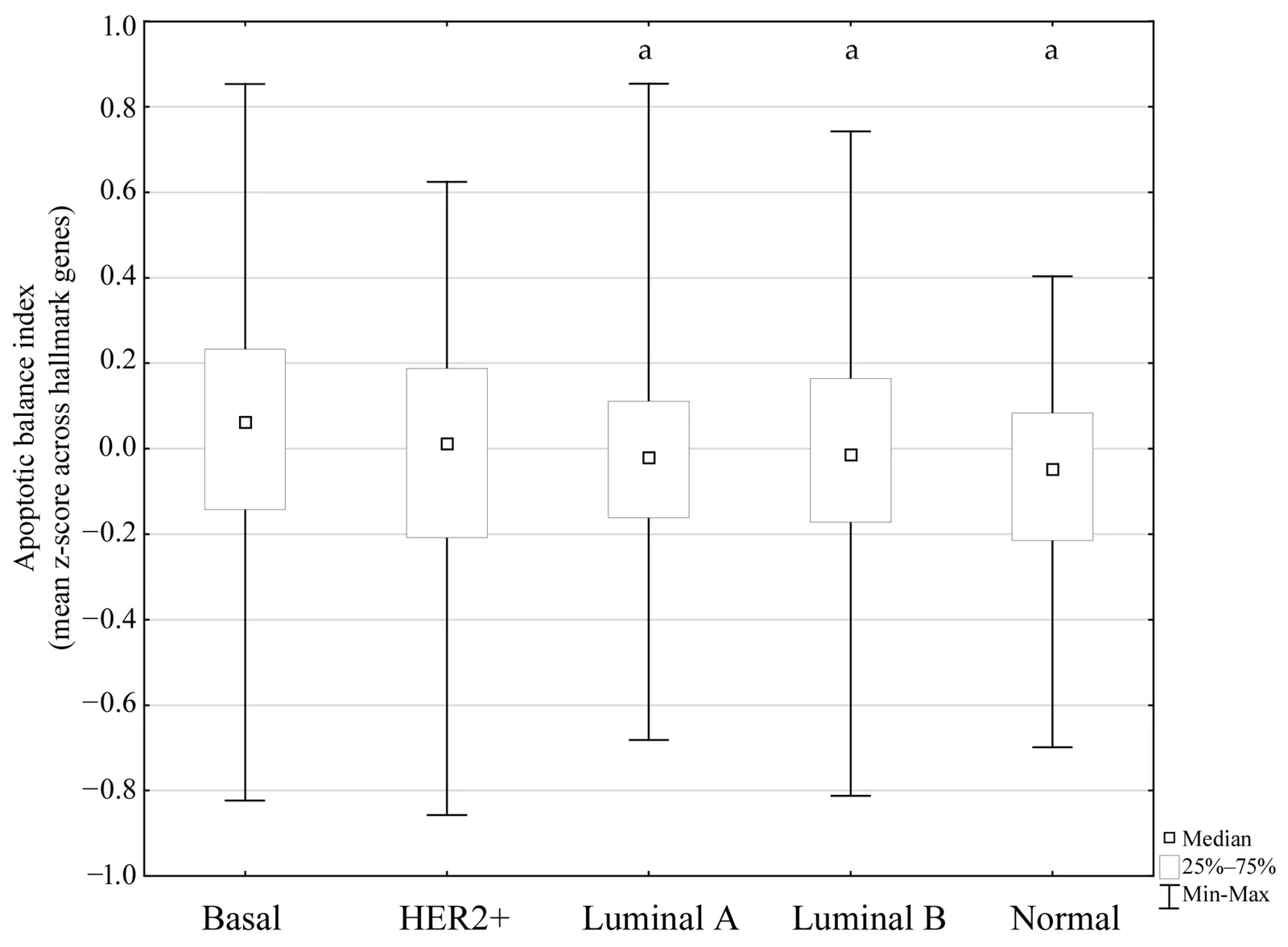
Apoptotic Balance Index (ABI) across PAM50 molecular subtypes in the METABRIC cohort. HER2—human epidermal growth factor receptor 2. ^a^ *p* < 0.05 vs. basal.

For comparability with the original classification, PAM50 molecular subtypes were used and harmonized into the following groups: luminal A (700 samples), luminal B (475 samples), HER2+ (224 samples), and basal (used as a proxy for TNBC, 209 samples). 148 normal-like samples were retained as a separate group, while claudin-low tumors were excluded due to their transcriptional heterogeneity and limited clinical annotation consistency. As shown in Figure 4, ABI demonstrated subtype-dependent differences across the METABRIC cohort, with basal-like tumors showing significantly distinct ABI values compared with luminal A and normal-like tissues. No consistent differences were observed among the remaining intermediate subtypes, which displayed partially overlapping distributions. Detailed statistical comparisons for the METABRIC validation cohort are provided in Supplementary Table S2.

These findings indicate that ABI captures a pronounced separation of basal-like breast cancer from luminal and normal-like tissues, rather than a uniform stratification across all molecular subtypes. Despite cohort-specific variations in intermediate groups, ABI consistently reflects biologically meaningful differences in apoptotic balance across breast cancer subtypes.

### 2.3. Identification of Key Apoptosis-Related Genes Associated with Apoptotic Balance Index Analysis

To identify specific genes contributing most strongly to the observed alterations in apoptotic balance, Spearman correlations were calculated between expression levels of HALLMARK_APOPTOSIS genes and ABI across all samples. The full correlation results for all HALLMARK_APOPTOSIS genes are provided in Supplementary Table S3. Genes demonstrating the strongest and most consistent correlations with ABI, while preserving expected biological function (pro- vs. anti-apoptotic), were selected for downstream validation. *CASP1*, previously included in an ongoing study, was excluded from the final list.

The final top five genes (*p* < 0.05) included two anti-apoptotic genes, *PPT1* (Spearman rho, −0.88) and *HSPB1* (Spearman rho, −0.85), and three pro-apoptotic genes, *BIK* (Spearman rho, 0.87), *TXNIP* (Spearman rho, 0.86), and *BMF* (Spearman rho, 0.86). The direction of correlation aligns with their expected functional roles: higher expression of anti-apoptotic genes was associated with lower ABI, whereas higher expression of pro-apoptotic genes correlated with increased ABI.

These findings highlight the selected genes as key modulators of apoptosis dysregulation in breast cancer and provide a rational basis for their subsequent validation at the mRNA and protein levels, as well as for exploration of their clinical significance in survival analyses.

### 2.4. Validation of Apoptosis-Related Gene Expression by qRT-PCR

To experimentally validate the transcriptomic findings, qRT-PCR was performed for five apoptosis-related genes (*BIK*, *BMF*, *TXNIP*, *HSPB1*, and *PPT1*) selected based on their strong correlations with the apoptotic balance index and pathway activity scores.

qRT-PCR analysis confirmed the direction of expression changes observed in the microarray dataset for all investigated genes, demonstrating consistent directional agreement between both platforms (Table 1).

The most pronounced and statistically significant expression changes were observed in TNBC, where all five genes exhibited expression levels significantly different from control tissues and from other molecular subtypes. Pro-apoptotic genes *BIK* and *BMF* displayed significantly reduced expression in TNBC and non-luminal HER2-positive tumors compared with control samples and luminal subtypes, consistent with suppressed intrinsic apoptotic signaling in aggressive breast cancer phenotypes. In contrast, anti-apoptotic genes *HSPB1* and *PPT1* were significantly upregulated in TNBC, indicating enhanced activation of survival-promoting mechanisms.

These subtype-specific expression patterns align with the observed decrease in intrinsic apoptotic pathway activity and apoptotic balance index in TNBC, supporting the functional relevance of the selected genes in apoptosis dysregulation associated with tumor aggressiveness.

### 2.5. Protein-Level Validation of Selected Apoptosis-Related Genes by ELISA

To further validate the transcriptomic findings at the protein level, ELISA was performed to quantify the concentrations of BIK, BMF, TXNIP, HSPB1, and PPT1 proteins in breast tumor and control tissue samples. ELISA results largely mirrored the gene expression patterns observed in both microarray and qRT-PCR analyses (Table 2).

In particular, significantly reduced protein levels of the pro-apoptotic factors BIK and BMF were detected in TNBC and non-luminal HER2-positive tumors compared with control tissues. Conversely, anti-apoptotic proteins HSPB1 and PPT1 showed significantly elevated levels in TNBC, consistent with enhanced survival signaling in aggressive breast cancer subtypes.

### 2.6. Identification of miRNAs Potentially Regulating Key Apoptosis-Related Genes

To investigate potential post-transcriptional regulation of key apoptosis-related genes, miRNA expression profiling was integrated with in silico target prediction for selected candidates derived from the HALLMARK_APOPTOSIS gene set. Differentially expressed miRNAs across breast cancer molecular subtypes were then matched with predicted target interactions to construct putative miRNA–mRNA regulatory relationships (Table 3).

Among the analyzed targets, BMF showed the most consistent evidence supporting potential miRNA-mediated regulation. *BMF* expression progressively decreased across increasing breast cancer aggressiveness, while its predicted targeting miRNAs (miR-582-5p and miR-421) were significantly overexpressed in more aggressive subtypes. In addition, both miRNAs demonstrated significant inverse correlations with BMF expression across matched samples (Spearman rho ≈ −0.7, *p* < 0.001), supporting a coherent inverse expression pattern consistent with putative regulatory interactions.

Similarly, *TXNIP* exhibited decreased expression in aggressive breast cancer subtypes, accompanied by upregulation of several predicted targeting miRNAs, including miR-106b-5p, miR-20b-5p, miR-20a-5p, and miR-93-5p. These miRNAs also showed inverse correlations with *TXNIP* expression (Spearman rho ≈ −0.6, *p* < 0.001), although the relationship appeared less consistent compared to *BMF*, suggesting additional layers of regulation beyond miRNA-mediated effects.

Overall, integration of differential expression patterns, target prediction, and correlation analyses supports the presence of biologically coherent miRNA–mRNA relationships for selected apoptosis-related genes, with *BMF* emerging as the most robust candidate for potential miRNA-mediated regulation.

### 2.7. Systemic Apoptotic Response Following Cryoablation in Fibroadenoma Patients

To characterize systemic molecular responses induced by cryoablation, peripheral blood samples were collected from 34 fibroadenoma patients at seven time points: before the procedure (T0), 30–60 min (T1), 8–12 h (T2), 48–72 h (T3), 7 days (T4), 1 month (T5), and 3 months (T6) after treatment.

qRT-PCR analysis revealed time-dependent modulation of apoptosis-related gene expression following cryoablation, which was mirrored at the protein level as determined by ELISA (Table 4).

*BIK*, *BMF*, *TXNIP* exhibited transient upregulation shortly after cryoablation, whereas cytoprotective genes *HSPB1*, *PPT1* showed delayed or sustained induction, suggesting a coordinated systemic apoptotic response followed by activation of protective mechanisms. These transcriptional changes were confirmed at the protein level, indicating activation of apoptosis-related pathways following cryoablation.

## 3. Discussion

In this study, we performed an integrative transcriptomic and functional analysis of apoptosis-related signaling across breast cancer molecular subtypes and in a benign cryoablation model. Using pathway-level scoring and an apoptotic balance index (ABI), we observed subtype-associated differences in apoptosis-related signaling, with a progressive shift toward reduced pro-apoptotic activity in more aggressive breast cancer subtypes, most pronounced in TNBC and non-luminal HER2+ tumors. From the HALLMARK_APOPTOSIS gene set, five key regulators (*BIK*, *BMF*, *TXNIP*, *HSPB1*, and *PPT1*) were selected based on their association with ABI, consistent expression patterns across platforms, and biological relevance to distinct functional aspects of apoptotic regulation, including mitochondrial signaling, stress response, metabolic regulation, and cellular adaptation processes. Collectively, these genes captured the observed anti-apoptotic shift, with suppression of pro-apoptotic factors (*BIK*, *BMF*, *TXNIP*) and concomitant upregulation of stress-adaptive or cytoprotective genes (*HSPB1*, *PPT1*) in aggressive breast cancer subtypes. Rather than acting as isolated determinants, these genes were further interpreted within broader functional modules reflecting coordinated apoptotic regulation.

BCL2-interacting killer (BIK) and BCL2-modifying factor (BMF), both belonging to the BH3-only protein family and involved in mitochondrial apoptosis initiation [15,16,17,18,19,20,21,22], showed reduced expression in aggressive breast cancer subtypes, consistent with previous reports indicating that downregulation of BH3-only proteins contributes to impaired intrinsic apoptotic signaling in breast cancer progression [23,24,25,26,27,28]. In addition, post-transcriptional regulatory analysis suggested that the observed downregulation of BMF may be partially associated with upregulation of miR-582-5p and miR-421, especially in more aggressive subtypes. These observations are consistent with previous studies in breast cancer, particularly TNBC, where overexpression of miR-582-5p was associated with increased metastatic potential [29]. In turn, miR-421 has been reported to exhibit context-dependent roles, acting either as an oncogenic or tumor-suppressive miRNA depending on the biological setting [30,31,32].

Similarly, reduced thioredoxin interacting protein (TXNIP) expression aligns with literature describing its role in linking metabolic stress and redox imbalance to apoptosis induction, where its loss contributes to metabolic reprogramming in cancer [33,34,35,36,37,38,39]. Our findings demonstrated suppression of *TXNIP* expression in aggressive breast cancer subtypes, accompanied by upregulation of miRNAs predicted to target *TXNIP*, including miR-106b-5p, miR-20a-5p, miR-20b-5p, and miR-93-5p. This observation is consistent with previous studies reporting oncogenic roles of these miRNAs in breast cancer [40,41,42,43,44,45] and supports a potential multi-layered regulatory network contributing to apoptotic dysregulation.

Heat shock protein beta-1 (HSPB1) has been widely reported as a stress-inducible chaperone associated with apoptosis inhibition, cytoskeletal stabilization, and therapy resistance in breast cancer [46,47,48,49,50,51]. In agreement with these reports, we observed increased *HSPB1* expression in aggressive subtypes, particularly TNBC, suggesting enhanced activation of cytoprotective stress-response mechanisms. However, its function should be interpreted in a context-dependent manner, as HSPB1 may also participate in broader cellular stress adaptation rather than acting exclusively as an anti-apoptotic factor.

Similarly, palmitoyl-protein thioesterase 1 (PPT1) has emerged in recent literature as a regulator of lysosomal function, autophagy, and cellular stress responses, with growing evidence linking it to tumor progression and survival pathways [52,53,54,55]. However, its role in apoptosis is indirect and context-dependent, and therefore *PPT1* should not be considered a strictly anti-apoptotic gene [56,57,58]. Our data demonstrated *PPT1* upregulation in aggressive breast cancer subtypes, which may reflect enhanced lysosomal adaptation and metabolic flexibility in aggressive tumor phenotypes.

Taken together, these data indicate that aggressive breast cancer subtypes exhibit a coordinated shift in apoptotic balance characterized by suppression of intrinsic apoptotic signaling and enhancement of stress-adaptive and survival-associated signaling. This pattern is consistent with pathway-level dysregulation rather than discrete gene-driven causality, and is in line with previous systems biology studies emphasizing the network-based nature of apoptosis regulation in cancer.

In contrast, fibroadenoma patients undergoing cryoablation demonstrated a transient systemic molecular response characterized by short-term activation of pro-apoptotic genes followed by gradual normalization. Similar transient apoptotic and inflammatory responses have been reported in the literature following ablative procedures, where localized tissue injury induces systemic stress-response signaling. Our findings extend these observations by demonstrating coordinated modulation of apoptosis-related genes and proteins measured across multiple time points, consistent with systemic stress-response signaling following cryoablation.

Although the present study focused exclusively on female breast cancer, male breast cancer represents a clinically important entity characterized by delayed diagnosis, distinct hormonal profiles, and frequently more aggressive clinical presentation [59,60]. Emerging evidence suggests that apoptosis regulation and therapeutic vulnerabilities may differ between male and female breast tumors. Future studies evaluating subtype-specific apoptotic signaling in male breast cancer could therefore provide valuable insights into sex-related differences in apoptosis regulation and treatment response [61,62,63,64].

Limitations of the study should be acknowledged. First, unequal representation of less common subtypes (non-luminal HER2+ and TNBC) and restriction to a Polish population, which may affect generalizability. Second, the number of patients undergoing cryoablation was relatively small, and follow-up time was limited to three months for molecular analyses. Third, RNA isolated from bulk tumor tissue reflects a complex mixture of signals originating from malignant epithelial cells as well as stromal and immune components. Therefore, observed gene expression patterns may partially reflect differences in cellular composition across breast cancer subtypes and should be interpreted with caution. Although no deconvolution analysis or single-cell resolution data were incorporated, the ABI was derived from a predefined apoptosis-related gene set and computed using a consistent framework across cohorts, supporting the robustness of pathway-level inference. External validation in the METABRIC cohort confirmed a subtype-dependent structure of ABI; however, differences in subtype ordering between datasets indicate that apoptotic signaling exhibits context-dependent behavior across breast cancer cohorts. Accordingly, ABI should be interpreted as a relative measure of apoptotic balance rather than a universal linear indicator of tumor aggressiveness. Fourth, while ELISA-based validation supported the observed expression trends at the protein level, the analysis relied on standard curve-based quantification without an internal housekeeping protein control, which may limit cross-sample normalization precision. Finally, miRNA–mRNA regulatory interactions were inferred based on in silico target prediction and therefore require further experimental validation to confirm their biological relevance.

## 4. Materials and Methods

### 4.1. Study Design

The study was divided into two main stages. In the first stage, the study material consisted of tissues from five molecular subtypes of breast tumors and corresponding control samples. Genome-wide mRNA expression profiling was performed using microarray technology. Apoptosis-related genes were defined based on the HALLMARK_APOPTOSIS gene set from the Molecular Signatures Database (MSigDB) (Supplementary Table S4). For each sample, an apoptosis pathway activation score was calculated using a gene set–based approach. Subsequently, genes contributing most strongly to apoptotic signaling were identified based on their correlations with the apoptosis pathway activation score and effect sizes across molecular subtypes. Genes demonstrating strong and consistent contributions to pathway activity across multiple breast cancer subtypes were selected for further analysis. The expression of selected genes was validated using quantitative reverse transcription polymerase chain reaction (qRT-PCR), and corresponding protein levels were assessed using enzyme-linked immunosorbent assay (ELISA). In parallel, differentially expressed microRNAs were identified, and bioinformatics databases were used to predict miRNA–gene interactions potentially regulating apoptotic signaling.

In the second stage of the study, peripheral blood samples were collected from patients diagnosed with breast fibroadenoma scheduled for cryoablation (before and after the procedure). The expression of genes selected in the first stage was quantified using qRT-PCR, and circulating protein levels were measured by ELISA. Focusing on genes with consistent involvement in apoptotic signaling enabled the assessment of whether early molecular responses in peripheral blood following cryoablation reflect apoptosis-related pathways identified in breast cancer tissue.

### 4.2. Patients with Breast Cancer

The study material was collected from 405 patients divided into five molecular subtypes: luminal A (130 cases), luminal B HER2− (100 cases), luminal B HER2+ (96 cases), non-luminal HER2+ (36 cases), and TNBC (43 cases). All cases were classified as T1N0M0. A margin of healthy tissue collected during the procedure served as the control group. Detailed clinicopathological characteristics of the breast cancer cohort, including age distribution and molecular subtype classification, are summarized in Supplementary Table S1 and were previously described [12,13,14].

All tissues were rinsed with sterile PBS, transferred to a tube containing 1 mL of TRIzol reagent (Invitrogen Life Technologies, Carlsbad, CA, USA; cat. no. 15596026), and stored at −80 °C. Total RNA was isolated using TRIzol reagent, and the resulting RNA extracts were purified using the RNeasy Mini Kit (QIAGEN, Hilden, Germany; cat. no. 74104) and treated with deoxyribonuclease I (Fermentas International Inc., Burlington, ON, Canada; cat. no. 18047019). RNA integrity was assessed by 1% agarose gel electrophoresis, and its purity and concentration were measured spectrophotometrically.

### 4.3. Patients with Breast Fibroadenoma

34 patients diagnosed with breast fibroadenoma were recruited for the study. They were scheduled for cryoablation using the IceCure ProSense™ system according to the manufacturer’s protocol. The mean age of the patients was 35.87 ± 4.11 years, and the mean BMI was 26.15 ± 4.98 kg/m^2^. Peripheral blood samples were collected from each patient at seven time points: before the procedure (T0), 30–60 min after cryoablation (T1), 8–12 h after (T2), 48–72 h after (T3), 7 days after (T4), 1 month after (T5), and 3 months after (T6).

Total RNA from peripheral blood was extracted using the PAXgene Blood RNA Kit (Qiagen, Valencia, CA, USA, cat. no. 762174) according to the manufacturer’s protocol. Similar to breast tissue samples, the obtained RNA was further purified, and its quality and concentration were assessed electrophoretically and spectrophotometrically. For protein analysis, peripheral blood samples were collected into serum-separating tubes. Serum was prepared according to the manufacturer’s instructions and stored at −80 °C until ELISA analysis.

### 4.4. mRNA Microarray Profiling

HG-U133A 2.0 arrays (Affymetrix, Santa Clara, CA, USA) with the GeneChip™ 3′IVT PLUS kit (Thermo Fisher Scientific, Inc., Waltham, MA, USA; cat. no. 902416) were used for microarray profiling in tissue samples according to the manufacturer’s instructions.

### 4.5. Transcriptomic and Pathway-Based Analysis of Apoptosis

To characterize genes associated with apoptotic signaling, pathway activity was evaluated using the HALLMARK_APOPTOSIS gene set from the Molecular Signatures Database (MSigDB) (Supplementary Table S4). For each sample, log2-normalized expression values of all available hallmark apoptosis genes were extracted from the microarray dataset. Gene-wise z-scores were calculated across all samples, and an apoptosis pathway activation score was computed for each sample as the mean z-score across all hallmark genes. Differences in pathway activity between breast cancer molecular subtypes and control tissue were assessed using the Kruskal–Wallis test followed by Dunn’s post hoc tests with Benjamini–Hochberg correction.

To further dissect the mechanisms underlying apoptotic signaling, apoptosis-related genes were subdivided into intrinsic (mitochondrial) and extrinsic (death receptor-mediated) pathways based on Reactome database (R-HSA-109606, REACT_1059). For each sample, intrinsic and extrinsic apoptosis pathway activation scores were calculated using the same gene set–based approach as described above.

To assess the balance between pro- and anti-apoptotic signaling, an apoptotic balance index (ABI) was calculated. Pro-apoptotic and anti-apoptotic genes were defined based on curated functional annotations obtained from the NCBI Gene (Entrez Gene) database, with particular emphasis on Gene Ontology (GO) Biological Process terms describing positive or negative regulation of apoptotic processes. Only genes included in the HALLMARK_APOPTOSIS gene set were considered. For each sample, ABI was computed as the difference between the mean expression z-scores of pro-apoptotic and anti-apoptotic genes, according to the formula:

ABI = mean(Z_pro-apoptotic genes_) − mean(Z_anti-apoptotic genes_)

Higher ABI values indicate a shift toward pro-apoptotic signaling, whereas lower values reflect dominance of anti-apoptotic mechanisms. For external validation, ABI was additionally computed in the METABRIC cohort [65] using the same gene set and computational framework. Gene expression data were obtained as normalized microarray values and transformed using gene-wise z-score normalization. One gene (F2), not present in the METABRIC dataset, was excluded from the analysis to ensure consistency across cohorts.

In a subsequent step, genes contributing most strongly to apoptotic pathway activity were identified. For each gene included in the HALLMARK_APOPTOSIS gene set, correlations between gene expression levels and the apoptosis pathway activation score were calculated. In parallel, effect sizes of gene expression differences across molecular subtypes were determined.

Genes selected for downstream experimental validation and survival analysis were chosen from among those demonstrating strong and consistent contributions to apoptosis pathway activity across breast cancer cohort. Selection criteria included correlation with the apoptosis pathway activation score, effect size, and biological relevance. Only genes meeting these criteria were subjected to further validation and clinical outcome analyses.

### 4.6. Quantitative Reverse Transcription Polymerase Chain Reaction (qRT-PCR)

Selected genes identified through microarray-based pathway analysis were validated at the mRNA level using qRT-PCR. Analyses were performed using both breast tumor tissue samples and peripheral blood samples collected from patients with breast fibroadenoma before and after cryoablation.

qRT-PCR experiments were conducted using the SensiFast SYBR No-ROX One-Step Kit (Bioline, London, UK) according to the manufacturer’s protocol. Gene expression levels were calculated using the 2^–ΔΔCt^ method. β-actin (ACTB) was used as the endogenous control. Primer sequences used in the study were as follows (5′–3′): *BIK* (Forward: GGAGGTTCTTGGCATGACTGAC, Reverse: TGAGGCTCACGTCCATCTCGTC), *BMF* (Forward: CAGTGGCAACATCAAGCAGAGG, Reverse: GCAAGGTTGTGCAGGAAGAGGA), *HSPB1* (Forward: CTGACGGTCAAGACCAAGGATG, Reverse: GTGTATTTCCGCGTGAAGCACC), *PPT1* (Forward: GGCGTACTCCAAAGTTGTTCAGG, Reverse: CTGCCAAGAAGATGCTGTGGTTG), *TXNIP* (Forward: CAGCAGTGCAAACAGACTTCGG, Reverse: CTGAGGAAGCTCAAAGCCGAAC), *ACTB* (Forward: TCACCCACACTGTGCCCATCTACGA, Reverse: CAGCGGAACCGCTCATTGCCAATGG).

### 4.7. Protein Quantification by Enzyme-Linked Immunosorbent Assay (ELISA)

Protein concentrations of selected apoptosis-related targets were quantified using ELISA in both breast tumor tissue samples and serum obtained from peripheral blood specimens of patients with breast fibroadenoma collected before and after cryoablation.

Commercially available ELISA kits were used according to the manufacturer instructions. The following kits (MyBioSource, Inc., San Diego, CA, USA) were used: BIK (MBS9322381), BMF (MBS728159), HSPB1 (MBS2021127), PPT1 (MBS1608842), TXNIP (MBS760271). Absorbance was measured using a microplate reader, and protein concentrations were calculated based on standard curves generated for each assay.

### 4.8. Global Profiling of Apoptosis-Related miRNAs and Target Prediction

To investigate microRNAs potentially involved in the regulation of apoptosis-related gene expression, miRNA profiling was performed using the GeneChip™ miRNA 2.0 Array (Affymetrix, Santa Clara, CA, USA) in breast tumor and corresponding control tissue samples. All experimental procedures were carried out in accordance with the manufacturer’s instructions.

To explore potential regulatory relationships between miRNAs and apoptosis-related genes, in silico target prediction analyses were conducted for the selected key genes using miRDB 6. 0 (http://mirdb.org, accessed on 15 December 2025). Only miRNA–mRNA interactions with high prediction confidence (target score > 80) were considered reliable [66].

### 4.9. Statistical Analysis

Statistical analyses were carried out using Transcriptome Analysis Console (Thermo Fisher Scientific, Waltham, MA, USA) and Statistica 13.3 (StatSoft, Krakow, Poland). The Shapiro–Wilk test was used to assess the data distribution, followed by the Kruskal–Wallis test and Dunn’s post hoc test with Benjamini–Hochberg correction. Correlations between gene expression levels and apoptotic balance index were calculated on a per-sample basis using Spearman’s rank correlation. Wilcoxon signed-rank test was used for paired longitudinal comparisons in patients undergoing cryoablation for fibroadenoma (T0 vs. follow-up), with Benjamini–Hochberg correction applied across all tested endpoints.

Sample sizes were calculated using G*Power 3.1.9.718 [67] based on a one-way analysis of variance (ANOVA) model, which served as an approximation for group-level comparisons. For the 405 patients recruited in this study, post hoc power analysis indicated a statistical power of 0.99.

The distribution of breast cancer subtypes based on national epidemiological data [68,69] was compared with that in our cohort. The proportions reported in the literature include luminal A (23.7%), luminal B HER2– (38.8%), luminal B HER2+ (14%), HER2+ (11.2%), and TNBC (12.3%). These values correspond to the subtype distribution in our cohort.

## 5. Conclusions

Our findings demonstrate subtype-associated differences in apoptosis-related signaling across breast cancer, with aggressive subtypes, particularly TNBC and non-luminal HER2+ tumors, exhibiting a coordinated shift toward reduced pro-apoptotic and enhanced stress-adaptive signaling. The apoptotic balance index (ABI) provided an integrative pathway-level measure capturing these differences and was supported by external validation in the METABRIC cohort.

Among HALLMARK_APOPTOSIS genes, *BIK*, *BMF*, *TXNIP*, *HSPB1*, and *PPT1* emerged as key apoptosis-associated regulators representing distinct functional modules related to mitochondrial apoptosis, metabolic regulation, stress response, and cellular adaptation. Integration of transcriptomic, protein-level, and miRNA-associated analyses supported biologically coherent patterns of apoptosis dysregulation across breast cancer subtypes.

In contrast, cryoablation of benign fibroadenomas was associated with transient systemic modulation of apoptosis-related pathways, consistent with regulated tissue stress responses. Collectively, these findings support the utility of pathway-level approaches for investigating apoptosis-related signaling and identify candidate molecular networks warranting further mechanistic and translational investigation in breast cancer.

## Figures and Tables

**Table 1:** Microarray and qRT-PCR validation of selected apoptosis-related genes across breast cancer subtypes.

Gene	Subtype vs. Control	Fold Change (log2)	
		Microarray	qRT-PCR
*BIK*	Luminal A	−1.52	−1.11
	Luminal B HER2−	−1.48	−1.25
	Luminal B HER2+	−1.92	−1.22
	Non-luminal HER2+	−2.51	−2.36
	TNBC	−5.32 ^a,b,c,d,e^	−3.92 ^a,b,c,d,e^
*BMF*	Luminal A	−1.36	−1.03
	Luminal B HER2−	−1.62	−1.33
	Luminal B HER2+	−2.41	−1.94
	Non-luminal HER2+	−4.07 ^a,b^	−2.11 ^a^
	TNBC	−5.33 ^a,b,c,d,e^	−4.45 ^a,b,c,d,e^
*HSPB1*	Luminal A	0.24	0.77
	Luminal B HER2−	0.9	0.83
	Luminal B HER2+	1.19	1.04
	Non-luminal HER2+	2.22 ^a,b^	2.41 ^a,b^
	TNBC	4.74 ^a,b,c,d,e^	3.88 ^a,b,c,d,e^
*PPT1*	Luminal A	1.21	1.05
	Luminal B HER2−	1.54	1.36
	Luminal B HER2+	1.42	1.51
	Non-luminal HER2+	2.8 ^a,b^	2.64 ^a,b^
	TNBC	4.22 ^a,b,c,d,e^	3.79 ^a,b,c,d,e^
*TXNIP*	Luminal A	−2.91	−1.52
	Luminal B HER2−	−2.36	−1.88
	Luminal B HER2+	−2.71	−1.93
	Non-luminal HER2+	−7.46 ^a^	−4.8 ^a^
	TNBC	−8.78 ^a,b,c,d,e^	−6.12 ^a,b,c,d,e^

HER2, human epidermal growth factor receptor 2; TNBC, triple-negative breast cancer; *BIK*, BCL2-interacting killer; *BMF*, BCL2-modifying factor; *HSPB1*, heat shock protein beta-1; *PPT1*, palmitoyl-protein thioesterase 1; *TXNIP*, thioredoxin interacting protein ^a^ *p* < 0.05 vs. control; ^b^ *p* < 0.05 vs. luminal A cancer, ^c^ *p* < 0.05 vs. luminal B HER2−, ^d^ *p* < 0.05 vs. luminal B HER2+, ^e^ *p* < 0.05 vs. non-luminal HER2+ cancer.

**Table 2:** Concentration of BIK, BMF, TXNIP, HSPB1, and PPT1 in breast cancer subtypes and control group (*p* < 0.05).

Protein [ng/mL]	Control	Luminal A	Luminal B HER2−	Luminal B HER2+	Non-Luminal HER2+	TNBC
BIK	4.12 ± 0.3	3.27 ± 0.2	2.99 ± 0.3	2.88 ± 0.2	1.77 ± 0.4 *	1.17 ± 0.3 *
BMF	3.67 ± 0.2	3.15 ± 0.3	3.01 ± 0.2	1.79 ± 0.3	1.02 ± 0.3 *	below detection threshold *
HSPB1	7.22 ± 0.3	9.74 ± 0.4	10.33 ± 0.3	11.04 ± 0.3	15.45 ± 0.4 *	19.83 ± 0.3 *
PPT1	5.65 ± 0.4	7.17 ± 0.3	7.66 ± 0.2	7.47 ± 0.2	8.11 ± 0.3	11.49 ± 0.3 *
TXNIP	3.12 ± 0.2	2.93 ± 0.2	2.76 ± 0.3	2.02 ± 0.4	below detection threshold *	below detection threshold *

HER2, human epidermal growth factor receptor 2; TNBC, triple-negative breast cancer; BIK, BCL2-interacting killer; BMF, BCL2-modifying factor; HSPB1, heat shock protein beta-1; PPT1, palmitoyl-protein thioesterase 1; TXNIP, thioredoxin-interacting protein. * *p* < 0.05 vs. control.

**Table 3:** Expression of miRNAs potentially involved in the regulation of studied genes.

Gene	miRNA	Fold Change (log2)						Spearman’s Correlation	
		Target Score	Luminal A vs. C	Luminal B HER2− vs. C	Luminal B HER2+ vs. C	Non-Luminal HER2+ vs. C	TNBC vs. C	Rho	*p*-Value
*BMF*	miR-582-5p	85	1.33	1.38	1.27	2.72 *	3.18 *	−0.77	*p* < 0.001
	miR-421	81	−1.09	1.14	1.82 *	1.98 *	2.05 *	−0.68	*p* < 0.001
*TXNIP*	miR-106b-5p	99	1.02	1.08	1.22	1.45	3.44 *	−0.66	*p* < 0.001
	miR-20b-5p	99	−1.1	1.17	1.35	1.51	3.03 *	−0.59	*p* < 0.001
	miR-20a-5p	99	1.25	1.55	1.69	1.62	2.56 *	−0.61	*p* < 0.001
	miR-93-5p	99	1.07	1.55	2.02 *	2.16 *	2.41 *	−0.64	*p* < 0.001

HER2, human epidermal growth factor receptor 2; TNBC, triple-negative breast cancer; C, control; *BMF*, BCL2-modifying factor; *TXNIP*, thioredoxin-interacting protein. * *p* < 0.05 vs. control.

**Table 4:** Changes in apoptosis-related gene expression and protein level in peripheral blood following cryoablation.

qRT-PCR Results							
Gene	Fold Change (log2)						
	T1 vs. T0	T2 vs. T0	T3 vs. T0		T4 vs. T0	T5 vs. T0	T6 vs. T0
*BIK*	1.15	2.21 *	1.65		1.20	1.12	1.05
*BMF*	1.40	2.60 *	1.90		1.25	1.20	1.02
*HSPB1*	−0.11	1.01	1.60		2.19 *	1.47	1.13
*PPT1*	1.02	1.09	1.38		1.92 *	1.35	1.18
*TXNIP*	1.93*	2.89 *	1.42		1.44	1.28	1.07
**ELISA results**							
Protein [ng/mL]	T0 (baseline)	T1 (after 30–60 min)	T2 (after 8–12 h)	T3 (after 48–72 h)	T4 (after 7 days)	T5 (after 1 month)	T6 (after 3 months)
BIK	2.55 ± 0.2	2.49 ± 0.3	2.71 ± 0.3	4.65 ± 0.2 *	2.63 ± 0.3	2.47 ± 0.4	2.59 ± 0.2
BMF	1.72 ± 0.3	1.85 ± 0.2	2.02 ± 0.4	4.05 ± 0.3 *	2.15 ± 0.2	1.79 ± 0.3	1.83 ± 0.4
HSPB1	9.09 ± 0.3	9.54 ± 0.2	9.28 ± 0.4	10.11 ± 0.2	16.46 ± 0.3 *	12.33 ± 0.3	9.21 ± 0.3
PPT1	3.47 ± 0.2	3.3 ± 0.4	3.72 ± 0.3	4.37 ± 0.4	7.07 ± 0.3 *	4.01 ± 0.3	3.66 ± 0.2
TXNIP	2.61 ± 0.3	2.58 ± 0.3	2.96 ± 0.3	5.42 ± 0.2 *	3.01 ± 0.4	2.87 ± 0.3	2.69 ± 0.3

*BIK*, BCL2-interacting killer; *BMF*, BCL2-modifying factor; *HSPB1*, heat shock protein beta-1; *PPT1*, palmitoyl-protein thioesterase 1; *TXNIP*, thioredoxin interacting protein. * *p* < 0.05 vs. control.

## Data Availability

The original contributions presented in this study are included in the article. The original contributions presented in the study are publicly available. These data can be found in the Figshare repository: Dysregulation of apoptotic signaling and apoptotic balance in breast cancer and benign cryoablation-induced tissue injury by Agata Panfil (2026). Figshare Dataset: https://doi.org/10.6084/m9.figshare.32542164. Further inquiries can be directed to the corresponding author.

## Supplementary Materials

The following supporting information can be downloaded at https://www.mdpi.com/article/10.3390/ijms27125174/s1.

## Abbreviations

The following abbreviations are used in this manuscript:

ABI	apoptotic balance index
BIK	BCL2-interacting killer
BMF	BCL2-modifying factor
HER2	human epidermal growth factor receptor 2
HSPB1	heat shock protein beta-1
PPT1	palmitoyl-protein thioesterase 1
TNBC	triple-negative breast cancer
TXNIP	thioredoxin-interacting protein
