# Supplementary material for: Apoptotic Signaling Across Breast Cancer Subtypes and Cryoablation-Induced Tissue Injury
Source: Int J Mol Sci. 2026 Jun 7;27(12):5174. doi: 10.3390/ijms27125174 (PMC13299198; doi:10.3390/ijms27125174)
Supplement: Supplementary file 1 [file ijms-27-05174-s001.zip › Supplementary Table S1.pdf]

Supplementary Table S1. Patient cohort characteristics.

Characteristics		Luminal A	HER2-negative luminal B	HER2-positive luminal B	Non-luminal HER2-positive	TNBC
**Age**	**< 50 years**	43 (33%)	32 (32%)	19 (20%)	9 (25%)	10 (23%)
	**> 50 years**	87 (67%)	68 (68%)	77 (80%)	27 (75%)	33 (77%)
**Grade**	**G1**	23 (18%)	31 (31%)	23 (24%)	9 (25%)	14 (32%)
	**G2**	48 (37%)	57 (57%)	57 (59%)	12 (33%)	21 (49%)
	**G3**	59 (45%)	12 (12%)	16 (17%)	15 (42%)	8 (19%)
**BMI [kg/m^2]**		30.78 ± 2.76	30.18 ± 4.56	32.09 ± 6.19	33.18 ± 5.67	34.67 ± 2.98

HER2, human epidermal growth factor receptor 2; TNBC, triple-negative breast cancer; BMI, body mass index.
