# Supplementary material for: Apoptotic Signaling Across Breast Cancer Subtypes and Cryoablation-Induced Tissue Injury
Source: Int J Mol Sci. 2026 Jun 7;27(12):5174. doi: 10.3390/ijms27125174 (PMC13299198; doi:10.3390/ijms27125174)
Supplement: Supplementary file 1 [file ijms-27-05174-s001.zip › Supplementary Table S2.pdf]

Supplementary Table S2. Apoptosis-related pathway activity across breast cancer subtypes.

Discovery cohort				
**Analysis**	**Comparison**	**Effect size**	**95% CI**	***p*-value**
Apoptosis Signaling Activity	HER2+ vs. Control	−0.31	[−0.54, −0.07]	0.04
	HER2+ vs. Luminal A	0.33	[0.11, 0.53]	0.04
	TNBC vs. Control	−0.24	[−0.39, −0.07]	0.02
	TNBC vs. Luminal A	−0.24	[−0.38, −0.10]	0.02
Intrinsic apoptosis<br>*BAX, BCL2L1, BCL2L11, BID, BMF, CASP3, CASP7, CASP9, DIABLO, PMAIP1, PPP3R1, XIAP*	HER2+ vs. Control	−0.81	[−0.85, −0.73]	< 0.001
	HER2+ vs. Luminal A	0.69	[0.54, 0.79]	0.005
	HER2+ vs. Luminal B HER2+	0.67	[0.48, 0.79]	0.04
	TNBC vs. Control	−0.62	[−0.71, −0.51]	< 0.001
	TNBC vs. Luminal A	−0.67	[−0.75, −0.58]	< 0.001
	TNBC vs. Luminal B HER2−	−0.49	[−0.60, −0.35]	< 0.001
	TNBC vs. Luminal B HER2+	−0.57	[−0.67, −0.44]	< 0.001
	TNBC vs. HER2+	−0.67	[−0.74, −0.58]	< 0.001
Extrinsic apoptosis<br>*CFLAR, FAS, FASLG, TNF, TNFSF10*	Luminal A vs. Control	−0.85	[−0.86, −0.78]	< 0.001
	Luminal B HER2− vs. Control	−0.82	[−0.85, −0.65]	< 0.001
	Luminal B HER2+ vs. Control	−0.85	[−0.86, −0.78]	< 0.001
	HER2+ vs. Control	−0.82	[−0.86, −0.73]	< 0.001
	TNBC vs. Control	−0.62	[−0.71, −0.51]	< 0.001
	TNBC vs. Luminal B HER2+	−0.24	[−0.40, −0.07]	0.02
	TNBC vs. HER2+	−0.32	[−0.46, −0.16]	< 0.001
Apoptotic balance index<br><br>Pro-apoptotic: *AIFM3, BAX, BCL2L11, BCL10, BID, BIK, BMF, CASP1, CASP3, CASP4, CASP6, CASP7, CASP8, CASP9, CYLD, DIABLO, FASLG, GADD45A, GADD45B, IGF2R, PDCD4, PMAIP1, RHOB, TNFRSF12A, TNFSF10, TOP2A, TXNIP*<br><br>Anti-apoptotic: *ANXA1 , BIRC3, CCND2 CD38, CD44, CFLAR, CLU, CTH, DFFA, DNAJC3, EGR3, ERBB2, ERBB3, F2R, HGF, HMOX1, HSPB1, IER3, IL1A, KRT18, LGALS3, MGMT, PEA15, PPT1, PTK2, SOD2, TIMP1, XIAP*	Luminal B HER2+ vs. Control	−0.85	[−0.86, −0.78]	0.02
	HER2+ vs. Control	−0.82	[−0.86, −0.73]	< 0.001
	HER2+ vs. Luminal A	0.85	[0.79, 0.86]	< 0.001
	TNBC vs. Control	−0.62	[−0.71, −0.51]	< 0.001
	TNBC vs. Luminal A	−0.68	[−0.75, −0.58]	< 0.001
	TNBC vs. Luminal B HER2−	−0.49	[−0.60, −0.35]	< 0.001
	TNBC vs. Luminal B HER2+	−0.57	[−0.67, −0.46]	< 0.001
	TNBC vs. HER2+	−0.74	[−0.80, −0.67]	< 0.001
**METABRIC validation**				
**Analysis**	**Comparison**	**Effect size**	**95% CI**	***p*-value**
Apoptotic balance index	Basal vs. Luminal A	0.13	[0.05, 0.19]	0.002
	Basal vs. Luminal B	0.10	[0.03, 0.18]	0.02
	Basal vs. Normal	0.19	[0.09, 0.28]	0.002
