# Supplementary material for: Apoptotic Signaling Across Breast Cancer Subtypes and Cryoablation-Induced Tissue Injury
Source: Int J Mol Sci. 2026 Jun 7;27(12):5174. doi: 10.3390/ijms27125174 (PMC13299198; doi:10.3390/ijms27125174)
Supplement: Supplementary file 1 [file ijms-27-05174-s001.zip › Supplementary Table S3.pdf]

Supplementary Table S3. Spearman correlations between expression levels of HALLMARK_APOPTOSIS genes and ABI across all samples.

Gene	Role	Spearman correlations	
		rho	*p*-value
PPT1	Anti-apoptotic	−0.88	< 0.001
CASP1	Pro-apoptotic	0.87	< 0.001
KRT18	Anti-apoptotic	0.87	< 0.001
BIK	Pro-apoptotic	0.87	< 0.001
TXNIP	Pro-apoptotic	0.86	< 0.001
BMF	Pro-apoptotic	0.86	< 0.001
F2R	Anti-apoptotic	0.86	< 0.001
HSPB1	Anti-apoptotic	−0.85	< 0.001
DIABLO	Pro-apoptotic	0.84	< 0.001
IER3	Anti-apoptotic	−0.84	< 0.001
HGF	Anti-apoptotic	−0.83	< 0.001
XIAP	Anti-apoptotic	−0.83	< 0.001
PMAIP1	Pro-apoptotic	0.82	< 0.001
CD38	Anti-apoptotic	−0.82	< 0.001
MGMT	Anti-apoptotic	0.82	< 0.001
CLU	Anti-apoptotic	−0.82	< 0.001
GADD45A	Pro-apoptotic	0.82	< 0.001
EGR3	Anti-apoptotic	0.81	< 0.001
TIMP1	Anti-apoptotic	−0.80	< 0.001
GADD45B	Pro-apoptotic	0.80	< 0.001
CASP3	Pro-apoptotic	0.80	< 0.001
CASP4	Pro-apoptotic	0.79	< 0.001
TOP2A	Pro-apoptotic	−0.78	< 0.001
CYLD	Pro-apoptotic	0.78	< 0.001
CFLAR	Anti-apoptotic	−0.78	< 0.001
BCL2L1	Anti-apoptotic	−0.78	< 0.001
CD44	Anti-apoptotic	−0.78	< 0.001
CASP6	Pro-apoptotic	0.77	< 0.001
PEA15	Anti-apoptotic	0.77	< 0.001
HMOX1	Anti-apoptotic	−0.77	< 0.001
CCND2	Anti-apoptotic	0.77	< 0.001
PDCD4	Pro-apoptotic	0.76	< 0.001
BAX	Pro-apoptotic	0.76	< 0.001
FASLG	Pro-apoptotic	0.76	< 0.001
ANXA1	Anti-apoptotic	−0.73	< 0.001
CTH	Anti-apoptotic	−0.73	< 0.001
CASP7	Pro-apoptotic	0.72	< 0.001
BCL10	Pro-apoptotic	−0.69	< 0.001
TNFRSF12A	Pro-apoptotic	−0.68	< 0.001
IGF2R	Pro-apoptotic	−0.66	< 0.001
PTK2	Anti-apoptotic	−0.65	< 0.001

BID	Pro-apoptotic	0.65	< 0.001
SOD2	Anti-apoptotic	−0.64	< 0.001
BCL2L11	Pro-apoptotic	−0.63	< 0.001
DNAJC3	Anti-apoptotic	0.61	< 0.001
DFFA	Anti-apoptotic	−0.59	< 0.001
IL1A	Anti-apoptotic	−0.56	< 0.001
ERBB3	Anti-apoptotic	0.48	< 0.001
ERBB2	Anti-apoptotic	−0.45	< 0.001
CASP9	Pro-apoptotic	0.33	< 0.001
BIRC3	Anti-apoptotic	−0.30	< 0.001
AIFM3	Pro-apoptotic	0.26	< 0.001
CASP8	Pro-apoptotic	−0.21	0.001
RHOB	Pro-apoptotic	0.20	0.002
TNFSF10	Pro-apoptotic	0.13	0.05
LGALS3	Anti-apoptotic	−0.12	0.06
