# Supplementary material for: Apoptotic Signaling Across Breast Cancer Subtypes and Cryoablation-Induced Tissue Injury
Source: Int J Mol Sci. 2026 Jun 7;27(12):5174. doi: 10.3390/ijms27125174 (PMC13299198; doi:10.3390/ijms27125174)
Supplement: Supplementary file 1 [file ijms-27-05174-s001.zip › Supplementary Table S4.pdf]

Supplementary Table S4. Members of the HALLMARK_APOPTOSIS human gene set from the Molecular Signatures Database.

Gene	NCBI (Entrez) Gene Id	Gene Symbol	Gene Description
ADD1	118	ADD1	adducin 1
AIFM3	150209	AIFM3	AIF family member 3
ANKH	56172	ANKH	ANKH inorganic pyrophosphate transport regulator
ANXA1	301	ANXA1	annexin A1
APP	351	APP	amyloid beta precursor protein
ATF3	467	ATF3	activating transcription factor 3
AVPR1A	552	AVPR1A	arginine vasopressin receptor 1A
BAX	581	BAX	BCL2 associated X, apoptosis regulator
BCAP31	10134	BCAP31	B cell receptor associated protein 31
BCL10	8915	BCL10	BCL10 immune signaling adaptor
BCL2L1	598	BCL2L1	BCL2 like 1
BCL2L10	10017	BCL2L10	BCL2 like 10
BCL2L11	10018	BCL2L11	BCL2 like 11
BCL2L2	599	BCL2L2	BCL2 like 2
BGN	633	BGN	biglycan
BID	637	BID	BH3 interacting domain death agonist
BIK	638	BIK	BCL2 interacting killer
BIRC3	330	BIRC3	baculoviral IAP repeat containing 3
BMF	90427	BMF	Bcl2 modifying factor
BMP2	650	BMP2	bone morphogenetic protein 2
BNIP3L	665	BNIP3L	BCL2 interacting protein 3 like
BRCA1	672	BRCA1	BRCA1 DNA repair associated
BTG2	7832	BTG2	BTG anti-proliferation factor 2
BTG3	10950	BTG3	BTG anti-proliferation factor 3
CASP1	834	CASP1	caspase 1
CASP2	835	CASP2	caspase 2
CASP3	836	CASP3	caspase 3
CASP4	837	CASP4	caspase 4
CASP6	839	CASP6	caspase 6
CASP7	840	CASP7	caspase 7
CASP8	841	CASP8	caspase 8
CASP9	842	CASP9	caspase 9
CAV1	857	CAV1	caveolin 1
CCNA1	8900	CCNA1	cyclin A1
CCND1	595	CCND1	cyclin D1
CCND2	894	CCND2	cyclin D2
CD14	929	CD14	CD14 molecule
CD2	914	CD2	CD2 molecule
CD38	952	CD38	CD38 molecule
CD44	960	CD44	CD44 molecule (IN blood group)
CD69	969	CD69	CD69 molecule
CDC25B	994	CDC25B	cell division cycle 25B

CDK2	1017	CDK2	cyclin dependent kinase 2
CDKN1A	1026	CDKN1A	cyclin dependent kinase inhibitor 1A
CDKN1B	1027	CDKN1B	cyclin dependent kinase inhibitor 1B
CFLAR	8837	CFLAR	CASP8 and FADD like apoptosis regulator
CLU	1191	CLU	clusterin
CREBBP	1387	CREBBP	CREB binding protein
CTH	1491	CTH	cystathionine gamma-lyase
CTNNB1	1499	CTNNB1	catenin beta 1
CYLD	1540	CYLD	CYLD lysine 63 deubiquitinase
DAP	1611	DAP	death associated protein
DAP3	7818	DAP3	death associated protein 3
DCN	1634	DCN	decorin
DDIT3	1649	DDIT3	DNA damage inducible transcript 3
DFFA	1676	DFFA	DNA fragmentation factor subunit alpha
DIABLO	56616	DIABLO	diablo IAP-binding mitochondrial protein
DNAJA1	3301	DNAJA1	DnaJ heat shock protein family (Hsp40) member A1
DNAJC3	5611	DNAJC3	DnaJ heat shock protein family (Hsp40) member C3
DNM1L	10059	DNM1L	dynamin 1 like
DPYD	1806	DPYD	dihydropyrimidine dehydrogenase
EBP	10682	EBP	EBP cholestenol delta-isomerase
EGR3	1960	EGR3	early growth response 3
EMP1	2012	EMP1	epithelial membrane protein 1
ENO2	2026	ENO2	enolase 2
ERBB2	2064	ERBB2	erb-b2 receptor tyrosine kinase 2
ERBB3	2065	ERBB3	erb-b2 receptor tyrosine kinase 3
EREG	2069	EREG	epiregulin
ETF1	2107	ETF1	eukaryotic translation termination factor 1
F2	2147	F2	coagulation factor II, thrombin
F2R	2149	F2R	coagulation factor II thrombin receptor
FAS	355	FAS	Fas cell surface death receptor
FASLG	356	FASLG	Fas ligand
FDXR	2232	FDXR	ferredoxin reductase
FEZ1	9638	FEZ1	fasciculation and elongation protein zeta 1
GADD45A	1647	GADD45A	growth arrest and DNA damage inducible alpha
GADD45B	4616	GADD45B	growth arrest and DNA damage inducible beta
GCH1	2643	GCH1	GTP cyclohydrolase 1
GNA15	2769	GNA15	G protein subunit alpha 15
GPX1	2876	GPX1	glutathione peroxidase 1
GPX3	2878	GPX3	glutathione peroxidase 3
GPX4	2879	GPX4	glutathione peroxidase 4
GSN	2934	GSN	gelsolin
GSR	2936	GSR	glutathione-disulfide reductase
GSTM1	2944	GSTM1	glutathione S-transferase mu 1
GUCY2D	3000	GUCY2D	guanylate cyclase 2D, retinal
H1F0	3005	H1-0	H1.0 linker histone
HGF	3082	HGF	hepatocyte growth factor
HMGB2	3148	HMGB2	high mobility group box 2
HMOX1	3162	HMOX1	heme oxygenase 1

HSPB1	3315	HSPB1	heat shock protein family B (small) member 1
IER3	8870	IER3	immediate early response 3
IFITM3	10410	IFITM3	interferon induced transmembrane protein 3
IFNB1	3456	IFNB1	interferon beta 1
IFNGR1	3459	IFNGR1	interferon gamma receptor 1
IGF2R	3482	IGF2R	insulin like growth factor 2 receptor
IGFBP6	3489	IGFBP6	insulin like growth factor binding protein 6
IL18	3606	IL18	interleukin 18
IL1A	3552	IL1A	interleukin 1 alpha
IL1B	3553	IL1B	interleukin 1 beta
IL6	3569	IL6	interleukin 6
IRF1	3659	IRF1	interferon regulatory factor 1
ISG20	3669	ISG20	interferon stimulated exonuclease gene 20
JUN	3725	JUN	Jun proto-oncogene, AP-1 transcription factor subunit
KRT18	3875	KRT18	keratin 18
LEF1	51176	LEF1	lymphoid enhancer binding factor 1
LGALS3	3958	LGALS3	galectin 3
LMNA	4000	LMNA	lamin A/C
LPPR4	9890	PLPPR4	phospholipid phosphatase related 4
LUM	4060	LUM	lumican
MADD	8567	MADD	MAP kinase activating death domain
MCL1	4170	MCL1	MCL1 apoptosis regulator, BCL2 family member
MGMT	4255	MGMT	O-6-methylguanine-DNA methyltransferase
MMP2	4313	MMP2	matrix metallopeptidase 2
NEDD9	4739	NEDD9	neural precursor cell expressed, developmentally down-regulated 9
NEFH	4744	NEFH	neurofilament heavy chain
PAK1	5058	PAK1	p21 (RAC1) activated kinase 1
PDCD4	27250	PDCD4	programmed cell death 4
PDGFRB	5159	PDGFRB	platelet derived growth factor receptor beta
PEA15	8682	PEA15	proliferation and apoptosis adaptor protein 15
PLAT	5327	PLAT	plasminogen activator, tissue type
PLCB2	5330	PLCB2	phospholipase C beta 2
PMAIP1	5366	PMAIP1	phorbol-12-myristate-13-acetate-induced protein 1
PPP2R5B	5526	PPP2R5B	protein phosphatase 2 regulatory subunit B'beta
PPP3R1	5534	PPP3R1	protein phosphatase 3 regulatory subunit B, alpha
PPT1	5538	PPT1	palmitoyl-protein thioesterase 1
PRF1	5551	PRF1	perforin 1
PSEN1	5663	PSEN1	presenilin 1
PSEN2	5664	PSEN2	presenilin 2
PTK2	5747	PTK2	protein tyrosine kinase 2
RARA	5914	RARA	retinoic acid receptor alpha
RELA	5970	RELA	RELA proto-oncogene, NF-kB subunit
RETSAT	54884	RETSAT	retinol saturase
RHOB	388	RHOB	ras homolog family member B
RHOT2	89941	RHOT2	ras homolog family member T2
RNASEL	6041	RNASEL	ribonuclease L

ROCK1	6093	ROCK1	Rho associated coiled-coil containing protein kinase 1
SAT1	6303	SAT1	spermidine/spermine N1-acetyltransferase 1
SATB1	6304	SATB1	SATB homeobox 1
SC5DL	6309	SC5D	sterol-C5-desaturase
SLC20A1	6574	SLC20A1	solute carrier family 20 member 1
SMAD7	4092	SMAD7	SMAD family member 7
SOD1	6647	SOD1	superoxide dismutase 1
SOD2	6648	SOD2	superoxide dismutase 2
SPTAN1	6709	SPTAN1	spectrin alpha, non-erythrocytic 1
SQSTM1	8878	SQSTM1	sequestosome 1
TAP1	6890	TAP1	transporter 1, ATP binding cassette subfamily B member
TGFB2	7042	TGFB2	transforming growth factor beta 2
TGFBR3	7049	TGFBR3	transforming growth factor beta receptor 3
TIMP1	7076	TIMP1	TIMP metallopeptidase inhibitor 1
TIMP2	7077	TIMP2	TIMP metallopeptidase inhibitor 2
TIMP3	7078	TIMP3	TIMP metallopeptidase inhibitor 3
TNF	7124	TNF	tumor necrosis factor
TNFRSF12A	51330	TNFRSF12A	TNF receptor superfamily member 12A
TNFSF10	8743	TNFSF10	TNF superfamily member 10
TOP2A	7153	TOP2A	DNA topoisomerase II alpha
TSPO	706	TSPO	translocator protein
TXNIP	10628	TXNIP	thioredoxin interacting protein
VDAC2	7417	VDAC2	voltage dependent anion channel 2
WEE1	7465	WEE1	WEE1 G2 checkpoint kinase
XIAP	331	XIAP	X-linked inhibitor of apoptosis
